# Supplementary material for: The limited screening accuracy of the Patient Health Questionnaire-2 in detecting depression among perinatal women in Italy
Source: PLoS One. 2021 Nov 29;16(11):e0260596. doi: 10.1371/journal.pone.0260596 (PMC8629231; doi:10.1371/journal.pone.0260596)
Supplement: Attachment — Submitted filename: Response to Reviewers.docx [file pone.0260596.s002.docx]

**Response to reviewers/Editor (responses are shown in blue)**

We address the reviewers/Editor concerns in this letter, and corresponding changes have been made to the manuscript.

1. Please ensure that your manuscript meets PLOS ONE's style requirements, including those for file naming. The PLOS ONE style templates can be found at 
https://journals.plos.org/plosone/s/file?id=wjVg/PLOSOne_formatting_sample_main_body.pdf and 
https://journals.plos.org/plosone/s/file?id=ba62/PLOSOne_formatting_sample_title_authors_affiliations.pdf

We have now revised the manuscript according to PLOS ONE's style requirements, including those for file naming.

2. Please review your reference list to ensure that it is complete and correct. If you have cited papers that have been retracted, please include the rationale for doing so in the manuscript text, or remove these references and replace them with relevant current references. Any changes to the reference list should be mentioned in the rebuttal letter that accompanies your revised manuscript. If you need to cite a retracted article, indicate the article’s retracted status in the References list and also include a citation and full reference for the retraction notice.

While revising your submission, please upload your figure files to the Preflight Analysis and Conversion Engine (PACE) digital diagnostic tool, <https://pacev2.apexcovantage.com/>. PACE helps ensure that figures meet PLOS requirements. To use PACE, you must first register as a user. Registration is free. Then, login and navigate to the UPLOAD tab, where you will find detailed instructions on how to use the tool. If you encounter any issues or have any questions when using PACE, please email PLOS at [figures@plos.org](mailto:figures@plos.org). Please note that Supporting Information files do not need this step.

We have reviewed our reference list to ensure that it was complete and correct. In the present and in the previous version of the manuscript we have not cited any papers which have been retracted.

Concerning figure files, we don’t have any figures.

3. PLOS ONE does not conduct masked peer review. Please add references into manuscripts.

We have now added those references.

4. In your Data Availability statement, you have not specified where the minimal data set underlying the results described in your manuscript can be found. PLOS defines a study's minimal data set as the underlying data used to reach the conclusions drawn in the manuscript and any additional data required to replicate the reported study findings in their entirety. All PLOS journals require that the minimal data set be made fully available. For more information about our data policy, please see http://journals.plos.org/plosone/s/data-availability.

Upon re-submitting your revised manuscript, please upload your study’s minimal underlying data set as either Supporting Information files or to a stable, public repository and include the relevant URLs, DOIs, or accession numbers within your revised cover letter. For a list of acceptable repositories, please see http://journals.plos.org/plosone/s/data-availability#loc-recommended-repositories. Any potentially identifying patient information must be fully anonymized.

Important: If there are ethical or legal restrictions to sharing your data publicly, please explain these restrictions in detail. Please see our guidelines for more information on what we consider unacceptable restrictions to publicly sharing data: http://journals.plos.org/plosone/s/data-availability#loc-unacceptable-data-access-restrictions. Note that it is not acceptable for the authors to be the sole named individuals responsible for ensuring data access.

We will update your Data Availability statement to reflect the information you provide in your cover letter.

We have now specified where the minimal data set can be found. We have upload our study’s minimal underlying data set as Supporting Information file.

Finally, in the Methods section of the revised manuscript we have now included all ethics information which were previously only entered in the system, at the time of first submission.
